# Overview of neuropathy associated with taxanes for the treatment of metastatic breast cancer

**DOI:** 10.1007/s00280-014-2607-5

**Published:** 2015-01-18

**Authors:** Edgardo Rivera, Mary Cianfrocca

**Affiliations:** Banner MD Anderson Cancer Center, 2946 E. Banner Gateway Drive, Gilbert, AZ 85234 USA

**Keywords:** Taxanes, Neuropathy, Metastatic breast cancer, *nab*-Paclitaxel, Docetaxel, Paclitaxel

## Abstract

Taxanes are an established option in the standard treatment paradigm for patients with metastatic breast cancer (MBC). Neuropathy is a common, dose-limiting side effect of taxane therapy that is often managed by dose reductions and delays. The severity, time to onset, and improvement in neuropathy are important considerations for patient management and vary among currently approved taxanes. The rate of grade ≥3 neuropathy with taxanes has been shown to be dose and schedule dependent; however, time to improvement to grade ≤1 is typically shorter for *nab*-paclitaxel than for other taxanes in patients with MBC. Many tools for assessing patient-reported neuropathy exist. Because MBC is incurable and patient quality of life must be critically considered when making treatment decisions, there is a need for more prospective trials to assess patient-reported neuropathy. Validated predictors of taxane-related neuropathy may play an important role in treatment decisions in the future. This review will focus on the toxicity profile (i.e., neuropathy) of each of the taxanes used in the treatment of MBC, will provide updates on tools used for the assessment of neuropathy, and will highlight newly discovered predictors of taxane-related neuropathy.

## Overview of taxane-related neuropathy

The National Comprehensive Cancer Network Guidelines recommend taxanes for the treatment of early-stage and metastatic breast cancer (MBC) [1]. Over the past several decades, the efficacy and safety profiles of taxanes—including paclitaxel (Taxol; Bristol-Myers Squibb), docetaxel (Taxotere; sanofi-aventis), and *nab*-paclitaxel (Abraxane; Celgene Corporation)—have been well established in MBC [2–4]. Neuropathy, a common side effect of taxane therapy, is a significant challenge for clinicians and patients. Taxane-associated neuropathy can compromise dose delivery and result in dose delays, reductions, or discontinuations that ultimately adversely affect treatment outcomes [5]. Some patients experience painful and persistent taxane-associated neuropathy that affects their activities of daily living and overall quality of life [6].

Taxane-associated peripheral neuropathies generally comprise sensory or motor neuropathy, depending on the type of nerve fibers involved [7]. The mechanism of taxane-induced neuropathy has been reported in numerous reviews [8–10]; thus, this review will not go into great detail regarding this topic. Briefly, neurons rely on transport and communication spanning the distance between the cell body and axons. These processes depend on intact and functional cytoskeletal microtubules. The binding of taxanes to the β-tubulin subunit of microtubules results in stabilization of the microtubule and disruption of microtubule function [11]. It is believed that the inhibition of microtubule function affects the structure and function of neurons, resulting in clinically apparent neuropathy [10]. The degree of neuronal damage depends on several factors, such as agent, cumulative dose, and duration of therapy [6]. Because of their extended axon length, peripheral nerves may be especially sensitive to taxane-induced damage, and the permeability of the blood–nerve barrier also lends itself to greater exposure of the sensory nerves to taxanes [7]. Taxane-related neuropathy may fall into several categories, namely sensory and motor. Sensory neuropathy generally manifests as bilateral sensations in the toes and fingertips, such as numbness, tingling, and pain; allodynia and diminished reflexes can also occur [12]. Motor weakness with taxane therapy generally affects the extremities [12].

Another important consideration in the development of taxane-related neuropathy is the solvent used in formulation, a key difference among the currently available taxanes [2–4]. Paclitaxel is formulated with polyoxyethylated castor oil, or Cremophor^®^ EL (recently renamed Kolliphor^®^ EL), docetaxel is formulated with polysorbate 80 (or TWEEN^®^ 80), and *nab*-paclitaxel is solvent free, consisting of paclitaxel and human serum albumin at a concentration similar to the concentration of albumin in the blood [2–4]. These differences can result in variations in toxicity profiles because the solvents themselves have been associated with varying biological effects. With respect to neurotoxicity, in preclinical studies, axonal swelling, degeneration, and demyelination have been observed with Cremophor EL [13, 14]. Thus, the damage it induces may be related to the persistent neuropathy caused by Cremophor EL-based paclitaxel. Polysorbate 80 may also contribute to the neuropathy observed with docetaxel by resulting in degeneration of neuronal vesicles [15]. In clinical studies of patients with MBC, severe neuropathy associated with paclitaxel and docetaxel persisted longer after discontinuation of therapy compared with that associated with *nab*-paclitaxel [16–18]. Severity of taxane-related neuropathy is related to a number of factors, including dosing and administration, which will be discussed later in this review.

Peripheral neuropathy related to chemotherapy can limit treatment for many patients. In a retrospective cohort study of patients receiving docetaxel or paclitaxel for nonmetastatic breast cancer, it was observed that the cumulative dose delivered was significantly lower than the planned cumulative dose in patients who had a dose reduction/discontinuation due to taxane-related chemotherapy (*P* < .001) [19]. In addition, taxane-related neuropathy is often cumulative and can progress after each treatment cycle [20]. Cumulative and persistent neuropathy in patients is often linked with a decreased ability to receive later lines of therapy. As an example, in a phase III trial of patients with advanced non-small cell lung cancer, those receiving first-line paclitaxel plus carboplatin demonstrated increased rates of peripheral neuropathy from cycle 4 (20 %) to cycle 8 (43 %) [21]; the most common reason for not progressing to second-line therapy was residual grade 2/3 peripheral neuropathy.

Data regarding the long-term effects of taxane-related peripheral neuropathy are varied. One study in patients who were 1–13 years post-taxane therapy demonstrated that paclitaxel- and docetaxel-related peripheral neuropathy completely resolved in only 14 % after treatment discontinuation [22]. However, the symptoms of peripheral neuropathy in these patients were considered to be well tolerated. In a second study of patients who were 6 months to 2 years post-adjuvant taxane therapy, 81 % of patients evaluated still reported symptoms of peripheral neuropathy, with up to 27 % reporting severe symptoms in the hands and feet [23]. Chemotherapy-related peripheral neuropathy may also be predictive of the development of neuropathic pain. A survey of patients who had previously received paclitaxel treatment for breast cancer revealed that 27 % of those who developed paclitaxel-related neuropathy eventually developed neuropathic pain [24]. The study suggested that monitoring patients who develop peripheral neuropathy on taxane treatment is important, even after treatment is discontinued.

The effects of taxane-related neuropathy may differ from other microtubule-inhibiting agents used to treat MBC. To date, no published head-to-head studies of taxanes and single-agent eribulin or ixabepilone exist, but preclinical evidence may provide clues about the neuropathic effects of these agents. In a preclinical study, mice treated with ixabepilone or paclitaxel had significant deficits in nerve conduction parameters as well as degenerative changes in the pathology of dorsal root ganglia and sciatic nerves, whereas mice treated with eribulin mesylate did not experience significant effects on nerve conduction and experienced less frequent morphological effects [25]. These findings led the authors to conclude that, in mice, eribulin mesylate was associated with less neuropathy compared with paclitaxel or ixabepilone. A subsequent preclinical study demonstrated that paclitaxel was associated with additional deleterious effects in mice with preexisting paclitaxel-induced peripheral neuropathy, whereas the neuropathic effect with eribulin in these mice was limited [26].

## Neuropathy in clinical trials of taxanes in the treatment of metastatic breast cancer

The development of taxane-related neuropathy has been reported in numerous phase II/III clinical trials (Table 1). A major limitation of many studies is the inconsistent reporting of neuropathy data; most studies report only grade ≥3 neuropathy. Taxane-related neuropathy appears to be related to dose and/or schedule [6, 10, 27–29].

**Table 1:** Taxane-induced peripheral neuropathy incidence in phase II/III clinical trials of metastatic breast cancer

Study	Population (no.)	Taxane	Dosage and schedule	Sensory neuropathy, %		
				Grade 2	Grade 3	Grade 4
Albain et al. [81]	Metastatic, previously treated (521)	Paclitaxel	175 mg/m^2^ q3w	18	4–5	<1
Andersson et al. [82]	Metastatic or locally advanced, previously treated (139)	Docetaxel	100 mg/m^2^ q3w	19	31	0
Fountzilas et al. [83]	Metastatic, previously treated (131)	Paclitaxel (plus carboplatin)	175 mg/m^2^ q3w	NR	5^a^ (grade 3 or higher)	
	Metastatic, previously treated (134)	Docetaxel	75 mg/m^2^ q3w	NR	0^a^ (grade 3 or higher)	
	Metastatic, previously treated (133)	Paclitaxel	80 mg/m^2^ weekly	NR	8^a^ (grade 3 or higher)	
Gradishar et al. [17]	Metastatic, previously treated (229)	*nab*-Paclitaxel	260 mg/m^2^ q3w	NR	10	0
	Metastatic, previously treated (225)	Paclitaxel	175 mg/m^2^ q3w	NR	2	0
Gradishar et al. [16]	Metastatic first-line (76)	*nab*-Paclitaxel	300 mg/m^2^ q3w	NR	21	0
	Metastatic first-line (76)	*nab*-Paclitaxel	100 mg/m^2^ qw	NR	9	0
	Metastatic first-line (74)	*nab*-Paclitaxel	150 mg/m^2^ qw	NR	22	0
	Metastatic first-line (74)	Docetaxel	100 mg/m^2^ q3w	NR	12	0
Miles et al. [84]	Metastatic or locally advanced, previously treated (231)	Docetaxel	100 mg/m^2^ q3w	NR	2 (grade 3 or higher)	
Miller et al. [85]	Metastatic or locally advanced, previously treated (346)	Paclitaxel	90 mg/m^2^ weekly for 3 of 4 weeks	NR	17	<1
Rivera et al. [28]	Metastatic or locally advanced, previously treated (59)	Docetaxel	75 mg/m^2^ q3w	NR	10 (grade 3 or higher)	
	Metastatic or locally advanced, previously treated (59)	Docetaxel	35 mg/m^2^ weekly every 3 of 4 weeks	NR	5 (grade 3 or higher)	
Seidman et al. [29]	Metastatic, previously treated (225)	Paclitaxel	175 mg/m^2^ q3w	21	12	0
	Metastatic, previously treated (346)	Paclitaxel	80–100 mg/m^2^ weekly	21	24–30	<1
Valero et al. [86]	Metastatic, previously treated (131)	Docetaxel	100 mg/m^2^ q3w	58 (grades 1–4); 3 (grade 3 or higher)		
	Metastatic, previously treated (131)	Docetaxel (plus carboplatin)	75 mg/m^2^ q3w	46 (grades 1–4); 1 (grade 3 or higher)		
Winer et al. [30]	Metastatic, previously treated (158)	Paclitaxel	175 mg/m^2^ q3w	57 (grades 1–4); 7 (grade 3 or higher)		
	Metastatic, previously treated (156)	Paclitaxel	210 mg/m^2^ q3w	73 (grades 1–4); 19 (grade 3 or higher)		
	Metastatic, previously treated (155)	Paclitaxel	250 mg/m^2^ q3w	83 (grades 1–4); 33 (grade 3 or higher)		

*NR* not reported, *q3w* every 3 weeks, *qw* every week

^a^ Sensory versus motor not delineated

### Neuropathy in trials of paclitaxel

In a phase III study of paclitaxel (± trastuzumab) administered weekly versus every 3 weeks (q3w) for the first- or second-line treatment of MBC [Cancer and Leukemia Group B (CALGB) 9840], the weekly schedule produced a significantly higher overall response rate (ORR) versus the q3w schedule (42 vs 29 %; *P* = .004) and a longer median time to progression (TTP; 9 vs 5 months; *P* < .0001) and median overall survival (OS; 24 vs 12 months; *P* = .0092) [29]. The weekly dose was decreased from 100 to 80 mg/m^2^ because of a 30 % incidence of sensory neuropathy [29]. Both treatment arms had similar incidences of grade 2 neuropathy (21 %), but the weekly schedule of paclitaxel produced twice as much grade 3 sensory neuropathy (24 %) versus the q3w schedule (12 %) (*P* = .0046 for grade ≥2). In the same study, the weekly arm was associated with a significantly higher rate of grade 2 and 3 motor neuropathy (8 and 9 %, respectively) versus the q3w arm (4 and 5 %, respectively) (*P* = .013 for grade ≥2). CALGB Protocol 9342 assessed the efficacy and safety of three paclitaxel doses administered q3w: 175, 210, and 250 mg/m^2^ in patients with MBC who had received ≥1 prior chemotherapy regimen for metastatic disease [30]. No differences were observed in ORR between the arms (21–26 %; *P* = NS) or in median OS (11–14 months; *P* = NS), but the 250-mg/m^2^ dose demonstrated a slightly longer median TTP versus the 210- and 175-mg/m^2^ doses (4.9 vs 4.1 vs 3.9 months, respectively; *P* = .045). The incidence of grade 3/4 sensory and motor neuropathy was dose related. The 250-mg/m^2^ dose produced the highest rate of grade 3/4 sensory and motor neuropathy (33 and 14 %, respectively), followed by the 210-mg/m^2^ dose (19 and 11 %, respectively) and the 175-mg/m^2^ dose (7 and 5 %, respectively). Interestingly, the incidences of grade 1/2 sensory neuropathy appeared to be higher in the 175- and 210-mg/m^2^ arms than in the 250-mg/m^2^ arm [79 vs 57 vs 35 %, respectively; *P* = not reported (NR)], as did the incidences of grade 1/2 motor neuropathy (89 vs 83 vs 79 %, respectively; *P* = NR).

### Neuropathy in trials of docetaxel

A phase III study assessed the efficacy and safety of docetaxel 75 mg/m^2^ q3w versus docetaxel 35 mg/m^2^ weekly for 3 weeks followed by 1 week of rest (qw 3/4) in patients who had received ≥one prior chemotherapy regimen for metastatic disease [28]. The q3w schedule demonstrated a numerically higher ORR (36 vs 20 %; *P* = NR), a similar median progression-free survival (PFS) (5.7 vs 5.5 months; *P* = NS), and OS (18.3 vs 18.6 months; *P* = NS), but more grade 3/4 neuropathy versus the weekly schedule (10 vs 5 %; *P* = NR). In a phase II study of patients with MBC who had received ≥one prior chemotherapy regimen for metastatic disease, docetaxel 40 mg/m^2^ weekly versus docetaxel 100 mg/m^2^ q3w, respectively, demonstrated similar ORRs (34 vs 33 %; *P* = NR) and median TTPs (5.7 vs 5.3 months; *P* = NR) and a longer median OS (29.1 vs 20.1 months; *P* = NR) [31]. Again, the q3w schedule demonstrated a higher incidence of grade 3/4 neurotoxicity (17 vs 2 %; *P* = NR), and more patients discontinued treatment in the q3w arm versus the weekly arm because of neurotoxicity (12 vs 2 %; *P* = NR). In a phase III trial of patients with anthracycline-pretreated MBC, no differences were observed between docetaxel 36 mg/m^2^ weekly and docetaxel 100 mg/m^2^ q3w in ORR (25 vs 26 %; *P* = NR), median PFS (4.5 vs 5.1 months; *P* = NS), and OS (7.8 vs 9.9 months; *P* = NS); however, the median time to treatment failure was significantly longer in the q3w arm (3.2 vs 4.0 weeks; *P* = .015) [32]. Grade 3/4 motor neuropathy was observed in 6.5 % of patients in the q3w arm versus 1.3 % of patients in the weekly arm, and 2.6 % of patients withdrew as a result of motor neuropathy in the q3w arm versus none in the weekly arm.

### Neuropathy in trials comparing paclitaxel with docetaxel

In a phase III study of paclitaxel 175 mg/m^2^ q3w versus docetaxel 100 mg/m^2^ q3w in patients with MBC that had progressed after an anthracycline-based regimen, docetaxel produced a significantly longer median OS (15.4 vs 12.7 months; *P* = .03), a significantly longer median TTP (5.7 vs 3.6 months; *P* < .0001), and a numerically higher ORR (32 vs 25 %; *P* = NS) versus paclitaxel [33]. Docetaxel versus paclitaxel produced a higher incidence of grade 3/4 neurosensory toxicity (7 vs 4 %; *P* = .08) and neuromotor toxicity (5 vs 2 %; *P* = .001) and more all-grade neurosensory toxicity (64 vs 59 %; *P* = NR) and neuromotor toxicity (28 vs 13 %; *P* = NR). A greater percentage of patients treated with docetaxel versus paclitaxel discontinued therapy because of neurosensory toxicity (8 vs 4 %) and neuromotor toxicity (5 vs 1 %).

### Neuropathy in trials comparing *nab*-paclitaxel with other taxanes

In the phase III study of *nab*-paclitaxel 260 mg/m^2^ versus paclitaxel 175 mg/m^2^ (both q3w), as ≥first-line therapy for patients with MBC, *nab*-paclitaxel produced a significantly longer ORR (33 vs 19 %; *P* = .001) and a significantly longer median TTP (5.3 vs 3.9 months; *P* = .006) [17]. No grade 4 sensory or motor neuropathy was observed in either arm; however, grade 3 sensory neuropathy was observed in 10 % of patients treated with *nab*-paclitaxel versus 2 % treated with paclitaxel (*P* < .001). The higher rate of neuropathy with *nab*-paclitaxel was not unexpected, because the actual delivered paclitaxel dose was 49 % higher with *nab*-paclitaxel versus paclitaxel (mean ± SD: 85.13 ± 3.118 vs 57.02 ± 3.008 mg/m^2^ per week, respectively). The incidence of grade 3 neuropathy observed with *nab*-paclitaxel 260 mg/m^2^ q3w was lower than that reported with a similar dose and schedule of paclitaxel in the CALGB 9342 trial. In that trial, 32 % of patients who received paclitaxel 250 mg/m^2^ q3w experienced grade 3 sensory neuropathy [30].

A randomized phase II study compared the efficacy and safety of various doses of qw 3/4 and q3w *nab*-paclitaxel versus docetaxel q3w as first-line treatment for MBC [27]. *nab*-Paclitaxel 150 mg/m^2^ qw 3/4 resulted in the longest median OS (33.8 months) followed by *nab*-paclitaxel 300 mg/m^2^ q3w (27.7 months), docetaxel 100 mg/m^2^ q3w (26.6 months), and *nab*-paclitaxel 100 mg/m^2^ qw 3/4 (22.2 months) [16]. Furthermore, treatment with *nab*-paclitaxel 100 mg/m^2^ qw 3/4, 150 mg/m^2^ qw 3/4, and 300 mg/m^2^ q3w resulted in a significantly higher investigator-assessed ORR versus docetaxel (63, 74, and 46 % vs 39 %; *P* < .001 overall); independently assessed ORR was also higher with the *nab*-paclitaxel 100 mg/m^2^ qw 3/4, 150 mg/m^2^ qw 3/4, and 300 mg/m^2^ q3w arms versus the docetaxel arm (34, 36, and 28 % vs 26 %; *P* = NS) [27]. Nonsignificant differences in the incidence of neuropathy existed between the various doses/schedules of *nab*-paclitaxel and docetaxel [16]. No grade 4 neuropathy was reported, but *nab*-paclitaxel at 300 mg/m^2^ q3w and 150 mg/m^2^ qw 3/4 resulted in the highest rates of sensory neuropathy (21 and 22 % of patients, respectively) versus 12 % of patients in the docetaxel arm and 9 % of patients in the *nab*-paclitaxel 100 mg/m^2^ qw 3/4 arm (*P* = NS overall) [16].

### Neuropathy in recent trials comparing *nab*-paclitaxel combinations with other taxane combinations

Rates of neuropathy associated with taxanes or ixabepilone plus bevacizumab for the treatment of MBC from a large cooperative group trial were reported [34]. The phase III CALGB 40502 trial compared the efficacy and safety of *nab*-paclitaxel 150 mg/m^2^ qw 3/4 plus bevacizumab 10 mg/kg q2w with either paclitaxel 90 mg/m^2^ qw 3/4 or ixabepilone 16 mg/m^2^ qw 3/4, both combined with the same dose/schedule of bevacizumab. A protocol amendment made the use of bevacizumab optional after the withdrawal of Food and Drug Administration approval in March 2011, but 98 % of patients in the study received bevacizumab. The median PFS (primary endpoint) was not statistically significantly different between the *nab*-paclitaxel (*n* = 271) and paclitaxel arms (*n* = 283) (9.2 vs 10.6 months; *P* = .12) and neither was the median OS (27 vs 26 months; *P* = .92). Rates of grade 2 neuropathy were similar between the *nab*-paclitaxel and paclitaxel arms (27 % for both) as were rates of grade 4 neuropathy (1 vs <1 %). However, a higher incidence of grade 3 neuropathy was observed in patients receiving *nab*-paclitaxel versus those receiving paclitaxel (24 vs 16 %) leading to a significantly higher rate of grade ≥3 neuropathy for *nab*-paclitaxel versus paclitaxel (25 vs 16 %; *P* = .012). Complete results from this study are eagerly anticipated. Similarly, in a phase II study of three different doses/schedules of *nab*-paclitaxel (260 mg/m^2^ q3w, 260 mg/m^2^ q2w with filgrastim, or 130 mg/m^2^ qw) with bevacizumab (10 mg/kg q2w or 15 mg/kg q3w) in patients with MBC, the ORRs (primary endpoint) were 45, 41, and 46 %, respectively, and rates of grade ≥3 neuropathy were 33, 56, and 46 %, respectively [35]. These findings suggest that *nab*-paclitaxel plus bevacizumab is active in MBC; however, the optimal schedule/dose of *nab*-paclitaxel requires further evaluation.

### Time to onset and improvement of neuropathy

Most phase II/III studies of taxanes in MBC have not reported time to onset or improvement of neuropathy, which are important factors affecting treatment decisions. One major reason for this may be that many patients have a preexisting level of neuropathy at enrollment because of prior chemotherapy treatment; thus, it may be difficult to distinguish preexisting neuropathy from that brought on by therapy. In one study of weekly paclitaxel 80 mg/m^2^ in patients with MBC who received ≤2 prior chemotherapy regimens for metastatic disease, 69 % of patients developed neuropathy and 9 % of patients developed grade 3 neuropathy (no grade 4) [36]. The median time to onset of grade 2/3 neuropathy in that study was approximately 4.7 months. In another study of paclitaxel 200–250 mg/m^2^ q3w, symptoms of neuropathy occurred after an average of 1.7 cycles in 84 % of patients [37].

Rapid improvement in neuropathic symptoms may allow patients to resume treatment more quickly and is important to note when considering timing, choice, and dose/schedule of later lines of therapy. Studies of *nab*-paclitaxel in MBC have reported time to improvement in neuropathy, and it is generally faster than with paclitaxel or docetaxel. In the study by Forsyth et al. of paclitaxel 200–250 mg/m^2^ q3w in previously treated patients with MBC, follow-up data were obtained in 19 % of patients; peripheral neuropathy had improved or resolved in all of these patients 1–6 months after treatment stopped [37]. In the phase II study by Gradishar et al. of *nab*-paclitaxel (100 and 150 mg/m^2^ qw 3/4 and 300 mg/m^2^ q3w) versus docetaxel 100 mg/m^2^ q3w for the first-line treatment of MBC, the median time to improvement in grade 3 neuropathy to grade ≤2 was 20–22 days with *nab*-paclitaxel compared with 41 days with docetaxel [16]. In the phase III study of *nab*-paclitaxel (260 mg/m^2^ qw) versus paclitaxel (175 mg/m^2^ qw) in patients with MBC by Gradishar et al., the median time to improvement in grade 3 neuropathy to grade ≤2 was 22 days for *nab*-paclitaxel and 79 days for paclitaxel [18]. Because peripheral neuropathy associated with *nab*-paclitaxel treatment appears to improve more quickly than with treatment with either paclitaxel or docetaxel, patients previously receiving *nab*-paclitaxel who have seen improvement in their peripheral neuropathy theoretically could progress to later lines of therapy more quickly.

## Predictors of taxane-induced neuropathy

Recent studies have identified potential molecular predictors of taxane-induced neuropathy. In the Eastern Cooperative Oncology Group (ECOG) 5103 study of women with early-stage breast cancer treated with weekly paclitaxel (± bevacizumab) in the adjuvant setting, a genome-wide association study showed that single nucleotide polymorphisms (SNPs) in two genes—*RWDD3* and *TECTA*—were significantly associated with time to onset of neuropathy (*P* < 5 × 10^−7^) [38], but this association could not be confirmed in a recent study of Scandinavian patients with ovarian cancer [39]. Another genome-wide association study in the CALGB 40101 trial showed a SNP in the gene *FGD4* that was associated with the early onset of peripheral neuropathy in patients treated with paclitaxel [40]. In the same study, other SNPs in the *EPHA5* and *FZD3* genes were also identified as being potential risk factors for the onset and severity of peripheral sensory neuropathy. In another study, patients with *ABCB1* variants were potentially more likely to develop paclitaxel-induced peripheral neuropathy than were those with the wild-type allele (*P* = .09) [41]. An association between a GSTP1 polymorphism and docetaxel-induced peripheral neuropathy has also been identified [42]. Patients with the ^105^Ile/^105^Ile *GSTP1* genotype had a significantly greater risk of developing more severe docetaxel-induced peripheral neuropathy than did those with other *GSTP1* genotypes (*P* = .03). Finally, in breast cancer patients treated with paclitaxel, CYP2C8*3 status was significantly associated with an increased risk of paclitaxel-induced neuropathy (*P* = .006); each CYP2C3*8 allele approximately doubled a patient’s risk of developing grade ≥2 neuropathy (*P* = .004) [43].

Age, race, and comorbid conditions, such as diabetes, may also be associated with an increased risk of developing neuropathy. The results of the ECOG 5103 study showed a significant association between neuropathy and age (12.9 % increase with each 10 years; *P* = .004) and African-American race (*P* = 4.5 × 10^−11^) [38]. In an analysis of the ECOG 1199 study of patients with breast cancer who received adjuvant taxane-containing therapy, hyperglycemia and obesity were associated with an increased risk of neuropathy, and African-American race demonstrated a trend toward an increased risk of neuropathy with weekly paclitaxel [44]. Although age was associated with an increased risk of neuropathy in the ECOG 5103 study [38], this trend was not observed in the ECOG 1199 study [44]. In that study, the development of neuropathy was found not to be predictive of survival outcomes. Furthermore, approximately half of all patients with diabetes are at risk of developing diabetic peripheral neuropathy [45, 46]. It is well known that high blood glucose can damage peripheral nerves; thus, patients with diabetes who are treated with chemotherapy agents associated with peripheral neuropathy, such as taxanes, may be at greater risk for developing chemotherapy-related peripheral neuropathy. While data are limited, some reports have suggested an increased risk of chemotherapy-related peripheral neuropathy or a worsening of preexisting neuropathy in patients with diabetes [47, 48]. With respect to taxane-based therapy, in an exploratory analysis of a phase III trial in patients with advanced non-small cell lung cancer, those with diabetes who were treated with *nab*-paclitaxel plus carboplatin had a 4 % higher rate of grade ≥3 peripheral neuropathy (7 %) than did the intent-to-treat population (3 %), while patients with diabetes who were treated with paclitaxel plus carboplatin had a 12 % higher rate of grade ≥3 peripheral neuropathy (23 %) compared with the intent-to-treat population (11 %) [49, 50]. While this finding is interesting, because of the exploratory nature of this analysis, no conclusions can be definitively drawn about whether patients with diabetes are at increased risk of developing peripheral neuropathy with *nab*-paclitaxel or paclitaxel regimens.

## Management of neuropathy

Early recognition of the signs and symptoms of taxane-related neuropathy is critical for appropriate management and improved outcomes. Typically, taxane-related neuropathy is managed with dose delays and/or reductions. For example, it is recommended that, for patients experiencing grade 3 neuropathy, the dose of *nab*-paclitaxel should be held until resolution to grade 1 or 2, followed by a dose reduction for all subsequent doses of *nab*-paclitaxel [2]. A 20 % dose reduction is recommended for all subsequent courses in patients receiving paclitaxel who develop severe peripheral neuropathy, and discontinuation is recommended in patients receiving docetaxel who develop grade 3/4 neuropathy [3, 4]. Numerous studies of interventions for the management of neuropathy exist; however, for the most part, many of these interventions have not demonstrated a meaningful improvement in neuropathic symptoms, and some agents have even worsened symptoms of chemotherapy-induced neuropathy compared with placebo (Table 2) [51–56]. Several of these interventions have been covered in previous reviews of taxane-related neuropathy [12, 57]. This review will focus only on recent highlights in this area.

**Table 2:** Select recent clinical studies of agents used to manage chemotherapy-induced peripheral neuropathy

Agent/study	*N*	Study type/findings
Gabapentin		
Rao et al. [52]	115	Phase III, randomized, double-blind study; no benefit of gabapentin versus placebo
Duloxetine		
Lavoie Smith et al. [87]	231	Phase III, randomized, double-blind study; duloxetine significantly reduced neuropathic pain outcomes versus placebo (*P* = .003)
Amitriptyline		
Kautio et al. [51]	114	Randomized, double-blind study; no benefit of amitriptyline versus placebo
Lamotrigine		
Rao et al. [53]	125	Phase III, randomized, double-blind study; no benefit of lamotrigine versus placebo
Acetyl-l-carnitine		
Hershman et al. [54]	409	Phase III, randomized, double-blind study; nonsignificantly decreased neuropathic symptoms at week 12, but significantly increased symptoms by week 24 versus placebo (*P* = .01)
Omega-3 fatty acids		
Ghoreishi et al. [59]	69	Randomized, double-blind study; significantly decreased incidence of peripheral neuropathy with omega-3 fatty acid pearls (*P* = .029) and nonsignificantly decreased peripheral neuropathy severity versus placebo
Vitamin E		
Kottschade et al. [88]		
	207	Phase III, randomized, double-blind study; no significant difference in time to onset of neuropathy, dose reductions as a result of neuropathy, or incidence of grade ≥2 sensory neuropathy with vitamin E versus placebo
Calcium and magnesium		
Grothey et al. [89]	102	Phase III, randomized, double-blind study; calcium and magnesium significantly decreased the incidence of grade ≥2 sensory neuropathy versus placebo (*P* = .038); this trial was terminated early because of a decreased response rate in the calcium and magnesium arm of the CONcePT study, which was also terminated early [90]
Topical BAK gel		
Barton et al. [91]	208	Phase III, randomized, double-blind study; BAK demonstrated a nonsignificant improvement in symptoms of sensory neuropathy and a significant improvement in motor neuropathy versus placebo (*P* = .021)

*BAK* baclofen, amitriptyline, and ketamine, *CONcePT* Combined Oxaliplatin Neurotoxicity Prevention Trial

Results of the phase III CALGB 170601 study of duloxetine 60 mg daily, in patients who developed chemotherapy-induced neuropathic pain after taxane or oxaliplatin treatment, showed that the drug was significantly more effective than placebo at reducing taxane-related neuropathic pain symptoms (*P* = .003) [58]. Patients treated with duloxetine also experienced a significant decrease in the amount of pain that interfered with daily functioning compared with placebo (*P* = .01). Furthermore, 41 % of patients treated with duloxetine reported a decrease in numbness and tingling in the feet compared with only 23 % of patients treated with placebo (*P* = NR); this trend was not observed for numbness and tingling in the hands (*P* = NR). Although the safety profile of duloxetine is generally considered to be acceptable, it should not be administered with drugs that inhibit serotonin reuptake, CYP P450 2D6 substrates, warfarin, or nonsteroidal anti-inflammatory drugs.

Another study found that omega-3 fatty acids may offer protection against paclitaxel-induced peripheral neuropathy [59]. In that study, patients with breast cancer received omega-3 fatty acid pearls or placebo during paclitaxel treatment. For 1 month after treatment, the patients were evaluated by using the reduced total neuropathy score; 70 % of the group that received omega-3 fatty acids did not develop peripheral neuropathy (any grade) compared with 41 % of patients in the placebo group (*P* = .029); no significant difference in the severity of peripheral neuropathy was observed between the 2 groups.

Other agents are in the early stages of assessment for the treatment or prevention of chemotherapy-related peripheral neuropathy. A recent preclinical study demonstrated that coadministration of metformin protected mice against chemotherapy-induced peripheral neuropathy [60]. The study found that cisplatin treatment led to the loss of intraepidermal nerve fibers in the paws and that metformin prevented this phenomenon. A separate preclinical study demonstrated that concurrent administration of interleukin-6 and cisplatin, vincristine, or paclitaxel in rodents prevented electrophysical abnormalities associated with neuropathy as well as pathological changes in peripheral nerves [61]. Treatment with interleukin-6 did not appear to affect the antitumor activity of these agents or affect tumor growth. Whether or not these preclinical findings will translate to humans remains to be seen.

Nonpharmaceutical methods of neuropathy management are also being studied. A case report of a patient with esophageal carcinoma who developed grade 2 peripheral neuropathy with docetaxel plus cisplatin demonstrated that manual therapy (i.e., massage) completely resolved the patient’s neuropathic symptoms [62]. In uncontrolled studies, treatment with a noninvasive electro-analgesia device, referred to as “Scrambler” therapy, demonstrated benefit for painful chemotherapy-induced peripheral neuropathy [63]; however, the results of other studies have been mixed. In a randomized controlled study of patients with neuropathic pain, Scrambler therapy appeared to be more beneficial than guideline-based drug management at relieving chronic neuropathic pain as assessed with a visual analog scale (*P* < .0001) [64]. However, Scrambler therapy failed to show any significant difference from sham therapy in pain scores in a recent randomized double-blind study of patients with neuropathic pain [65]. Further studies of Scrambler therapy in various solid tumors are ongoing. Several case series, case reports, and other small studies have demonstrated improvement in neuropathic symptoms with acupuncture [66–69]. A phase II randomized trial that will assess the effects of acupuncture in preventing dose reductions due to chemotherapy-induced peripheral neuropathy in patients with breast cancer is currently ongoing (ClinicalTrials.gov identifier NCT01881932).

## Neuropathy assessment tools

Many times, neuropathy is underrecognized and underreported by physicians compared with patients [70–72]. Furthermore, neuropathy induced by taxanes is generally quantified in clinical studies by using toxicity grading scales [e.g., National Cancer Institute Common Toxicity Criteria for Adverse Events (NCI CTCAE)], and these scales often have suboptimal reliability, sensitivity, and validity [71, 73, 74]. Therefore, patient-reported neuropathy tools are important for assessing the development and improvement in chemotherapy-induced peripheral neuropathy, and more prospective studies using these types of tools in trials of MBC are critically needed. A major advantage to these patient-reported tools is the ability to capture treatment effects over time, even after treatment has been completed. This is important for patients receiving taxane therapy, because peripheral neuropathy with some taxanes can persist for an extended period of time.

Select patient-reported tools for use in assessing taxane-induced neuropathy are shown in Table 3. Numerous patient-reported tools have been validated [75]; however, the Functional Assessment of Cancer Therapy (FACT)-Taxane tool is the only taxane-specific tool for assessing patient-reported taxane-related symptoms, including neuropathy [76]. FACT-Taxane consists of a 16-item FACT-General (FACT-G) and an 11-item taxane subscale that allow for the evaluation of disease symptoms and taxane-related toxicity [77]. The FACT-Ntx scale is another iteration of the FACT family of tools that specifically measures neurotoxicity produced by chemotherapy [77]. The FACT-Ntx is an 11-item questionnaire that focuses solely on chemotherapy-induced neurotoxicity; it is commonly used in combination with the FACT-G scale as part of the FACT/Gynecologic Oncology Group Neurotoxicity scale. In one study, the addition of paclitaxel to cisplatin plus doxorubicin in patients with advanced endometrial cancer produced more neuropathy and lower FACT-Ntx scores (indicating worse neuropathy) compared with doxorubicin plus cisplatin alone (*P* < .001), and the differences were still significant after 6 months (*P* = .014) [78].

**Table 3:** Patient-reported tools used to assess peripheral neuropathy in clinical trials

Measure	Description
FACT/GOG-Ntx subscale [73, 74, 77, 92]	11-Item neurotoxicity subscale of FACT-General
	Scoring: 0–4, with 0 representing “not at all” and 4 representing “very much”
	Example item: I feel discomfort in my hands.
QLQ-CIPN20 [80]	20-Item self-report questionnaire designed to supplement the EORTC Quality Of Life Questionnaire
	Scoring: 1–4, with 1 representing “not at all” and 4 representing “very much.”
	Example item: during the past week, did you have tingling toes or feet?
Chemotherapy-Induced Peripheral Neuropathy Tool [79]	50-Item self-report questionnaire
	Scoring: 0–10, with 0 representing “not at all” and “never” and 10 representing “completely,” “always,” or “extremely”
	Example item: at its worst, how severe is the numbness in the hands?
Patient Neurotoxicity Questionnaire [70]	2 Subjective items that assess sensory and motor neuropathy
	Scoring: A (no neuropathy) to E (severe neuropathy)
	Example item: I have no numbness, pain, or tingling in my hands or feet.

*EORTC* European Organisation for the Research and Treatment of Cancer, *FACT* Functional Assessment of Cancer Therapy, *FACT/GOG-Ntx* FACT/Gynecologic Oncology Group Neurotoxicity, *QLQ*-*CIPN20* Quality of Life Questionnaire–Chemotherapy-Induced Peripheral Neuropathy 20

The Chemotherapy-Induced Peripheral Neuropathy Assessment Tool (CIPNAT) was recently developed but has not yet been validated in any studies to date [79]. The CIPNAT is a 36-item tool that evaluates the occurrence, severity, distress, and frequency of nine neuropathic symptoms and 14 items that evaluate neuropathic interference with activities. Another tool, the Patient Neurotoxicity Questionnaire (PNQ), contains only two items to assess the incidence and severity of sensory and motor neuropathy [70]. It was prospectively assessed in a phase III study of patients with breast cancer receiving adjuvant therapy with a taxane [70]. The PNQ scores were compared with FACT-Ntx, FACT-G, and NCI CTCAE scores, and the results showed a strong correlation between the PNQ and the FACT-Ntx but only a weak correlation between the PNQ and the FACT-G. The PNQ scores were significantly correlated with the NCI CTCAE sensory neuropathy scores but not with the motor neuropathy scores; the physician-assessed scores were lower than the patient-reported scores in this study. Finally, the Quality of Life Questionnaire (QLQ)–Chemotherapy-Induced Peripheral Neuropathy (CIPN) 20 scale is a 20-item patient-reported tool that supplements the European Organization for Research and Treatment of Cancer QLQ questionnaire [80]. The QLQ-CIPN20 recently demonstrated good validity and reliability scores in a standardization study performed by the Chemotherapy-Induced Peripheral Neuropathy Outcome Measures Study Group, and further studies are planned to evaluate the responsiveness aspects of this tool [73].

## Conclusions and future perspectives

Although newer targeted agents are being developed, taxanes remain a standard of care therapy for patients with MBC. Neuropathy is an important, dose-limiting, painful, and often irreversible toxicity associated with taxane therapy. Thus, there exists a need to balance taxane efficacy and toxicity. Fully understanding the differences in the development and improvement in neuropathy between the taxanes is highly important for making treatment decisions and proactively managing patients with MBC on taxane therapy.
